# Comparative efficacy of non-pharmacological interventions on fear of childbirth for pregnant women: a systematic review and network meta-analysis

**DOI:** 10.3389/fpsyg.2025.1530311

**Published:** 2025-03-12

**Authors:** Juan Zhou, Zhengting Zhu, Ruoyu Li, Xiujing Guo, Dehua Li

**Affiliations:** ^1^Department of Nursing, West China Second University Hospital, Sichuan University/West China School of Nursing, Sichuan University, Chengdu, China; ^2^Key Laboratory of Birth Defects and Related Diseases of Women and Children (Sichuan University), Ministry of Education, Chengdu, China

**Keywords:** non-pharmacological interventions, fear of childbirth, FOC, pregnant women, network meta-analysis

## Abstract

**Objective:**

To explore effectiveness of non-pharmacological interventions in fear of childbirth.

**Methods:**

All published literature were searched from three databases (Pubmed, Cochrane CENTRAL, and Web of Science) as of April 2024. The risk of bias of the included studies was assessed using the Cochrane Systematic Review Manual 2.0 bias risk assessment tool. The primary outcome was FOC, the secondary outcomes were depression, anxiety, stress, childbirth self-efficacy, and mode of delivery.

**Results:**

This study included 32 randomized controlled trials, involving 17 interventions and 3,187 pregnant women. Compared with usual care, cognitive-behavioral therapy (SMD = −1.62, 95%CI –2.47 to −0.66), haptonomy (SMD = −1.43, 95%CI –2.63 to −0.24), motivational interview (SMD = −1.35, 95%CI –2.35 to −0.35), counseling therapy (SMD = −1.08, 95%CI –1.91 to −0.25) statistically and significantly improved fear of childbirth in gestational period. Emotional freedom technique (SMD = −3.13, 95%CI –5.00 to −1.26), counseling therapy (SMD = −1.81, 95%CI –2.97 to −0.80), haptonomy (SMD = −1.78, 95%CI –2.89 to −0.66), cognitive-behavioral therapy (SMD = −1.42, 95%CI –2.53 to −0.32), motivational interview (SMD = −1.28, 95%CI –2.37 to −0.19) statistically and significantly improved fear of childbirth in postnatal period. The cluster analysis showed that emotional freedom technique, haptonomy, motivational interview, cognitive-behavioral therapy, counseling therapy were considered to be more effective non-pharmacological interventions.

**Conclusion:**

Several non-pharmacological interventions are promising in the daily care of pregnant women with fear of childbirth. Healthcare professionals should be encouraged to apply these non-pharmacological interventions for informal caregivers of pregnant women with fear of childbirth.

**Systematic Review Registration:**

https://www.crd.york.ac.uk/PROSPERO, CRD42024536944.

## 1 Introduction

Fear of childbirth (FOC), a prominent perinatal mental health challenge, has been shown to exert profound effects on both maternal and infant health outcomes (Chen et al., 2024). Defined as persistent anxiety and psychological distress associated with pregnancy and childbirth, FOC manifests through symptoms such as mood disorders, somatization, altered decision-making behaviors, and, in some cases, avoidance behaviors like opting for non-medically indicated cesarean sections or seeking active contraception (Alizadeh-Dibazari et al., 2024). Global epidemiological studies indicate that approximately 80% of pregnant women experience varying degrees of FOC, with 6.3 to 14.8% (Nilsson et al., 2018) meeting the diagnostic criteria for severe FOC, often classified as tokophobia. This specific fear disorder is significantly linked to an increased risk of postpartum depression, prolonged labor, and higher rates of neonatal NICU admissions (Horsch et al., 2024).

FOC has been found to be influenced by a range of factors, which can be categorized into four key domains: (1) psychological factors, such as anxiety, depression, stress, and low self-efficacy regarding childbirth (Dencker et al., 2019; Hildingsson et al., 2024; Zhang et al., 2023); (2) sociodemographic factors, including lower educational levels, economic disadvantage, and insufficient social support (Behmanesh et al., 2024; Dencker et al., 2019); (3) growth experiences and cultural perceptions, which encompass a history of emotional abuse or neglect during childhood, as well as cultural views that perceive childbirth as a high-risk medical event; and (Dencker et al., 2019; Porthan et al., 2023; Preis et al., 2018) (4) spiritual health, with higher levels of spiritual well-being being associated with a reduced risk of FOC (Behmanesh et al., 2024). Existing literature consistently reports that primigravid women tend to experience significantly higher levels of FOC compared to multiparous women (Badaoui et al., 2019; Behmanesh et al., 2024; Nieminen et al., 2017; Zhang et al., 2023).

FOC can trigger a cascade of stress responses in pregnant women, manifesting as physiological alterations including elevated heart rate, increased blood pressure, and heightened cortisol levels (Alder et al., 2011; Reissland and Kisilevsky, 2016). These physiological changes may induce uterine environmental modifications that elevate risks of intrauterine growth restriction, fetal distress, preterm delivery, and low birth weight infants (Van den Bergh et al., 2005). Concurrently, the emotional and psychological reactions associated with labor anxiety are clinically correlated with reduced breastfeeding intention and impaired postpartum role adaptation (Challacombe et al., 2021; Yin et al., 2024). Such emotional and psychological responses may adversely affect the establishment of early maternal–infant attachment (Seefeld et al., 2021) and ultimately compromise the physical and psychological development of the newborn.

FOC can significantly hinder the development of a maternal social support network (Zhang et al., 2023). It not only diminishes a woman’s positive expectations about childbirth but also influences the attitudes of her spouse and family towards the experience (Aguilera-Martín et al., 2021). Women experiencing FOC often feel isolated and reluctant to share their concerns with others. Furthermore, societal idealization of childbirth may lead to feelings of self-blame and shame among pregnant women, which can exacerbate their psychological distress (Nilsson et al., 2018).

FOC imposes a dual burden on both the healthcare system and the broader socio-economic landscape. Studies have demonstrated that women with severe FOC are more likely to seek frequent medical consultations for FOC-related symptoms, experience prolonged sick leave and postpartum hospitalization, and have higher rates of psychotropic medication use and a greater need for psychosocial interventions compared to those with lower levels of FOC (Nieminen et al., 2017). For instance, in Sweden, the average total social costs for women with severe FOC were 10,830.2 euros, which was 38% higher than the control group (Nieminen et al., 2017). Additionally, FOC is a significant driver of medically unindicated CS, with pregnant women requesting CS due to concerns such as distrust of healthcare providers, fear of labor pain (particularly when epidural analgesia is unavailable or unaffordable), fear of pelvic floor injuries and incontinence, an concerns about the potential negative impact on their sexuality or sexual relationships (Betrán et al., 2018). The global CS rate has increased from 7% in 1990 to 21% in 2018, with rates reaching as high as 50% in low- and middle-income countries, such as those in Asia and South America (Ana et al., 2021). In these regions, inadequate primary healthcare and pregnant women’s mistrust of healthcare workers often lead them to opt for cesarean delivery to avoid perceived poor-quality labor and delivery care (Etcheverry et al., 2024; Long et al., 2018). This overuse of CS is medically unjustified and results in the unnecessary consumption of healthcare resources (Betrán et al., 2018).

Non-pharmacological interventions are the preferred first-line treatments for FOC. Previous reviews have reported antenatal education, psychoeducation and midwifery counselling as effective interventions for reducing FOC (Akgün et al., 2020; Alizadeh-Dibazari et al., 2023; Alizadeh-Dibazari et al., 2024; Bakhteh et al., 2024). However, the evidence for the effectiveness of cognitive-behavioral therapy (CBT), mindfulness-based therapy and various newer alternative treatments remains unclear or conflicting (Aguilera-Martín et al., 2021; Badaoui et al., 2019; Neo et al., 2022; Striebich et al., 2018). Some meta-analyses included small sample sizes (Akgün et al., 2020; O'Connell et al., 2021) and incorporated non-randomized controlled trials (Akgün et al., 2020; Evans et al., 2022), which may compromise statistical power and evidence quality. Most studies (Aguilera-Martín et al., 2021; Alizadeh-Dibazari et al., 2024; O'Connell et al., 2021; Striebich et al., 2018) failed to measure psychological indicators closely related to FOC such as anxiety, depression, and childbirth self-efficacy, while also lacking follow-up assessments of postpartum and long-term psychological outcomes. Furthermore, most conventional meta-analyses only compared data between two interventions, failing to rank the effectiveness of multiple interventions. Consequently, it remains challenging to determine the optimal non-pharmacological intervention for reducing childbirth fear.

Network Meta-Analysis (NMA) is an advanced statistical method used to simultaneously compare the effects of multiple interventions, even when there are no direct comparative studies between them. By integrating both direct comparisons (e.g., A vs. B) and indirect comparisons (e.g., A vs. C inferred through A vs. B and B vs. C), NMA constructs a network structure. This allows for a unified ranking and comprehensive evaluation of all interventions (Dias and Caldwell, 2019).

This study is the first to apply NMA to systematically review and analyze existing evidence, including randomized controlled trials of non-pharmacological interventions for FOC. The study aims to assess the effectiveness of various non-pharmacological interventions on FOC and rank their effects. Additionally, it considers the effects of these interventions on CS rate and psychological outcomes closely related to FOC, such as anxiety, depression, stress, and childbirth self-efficacy. Our study addresses a gap in the existing literature and provides an evidence-based foundation for enhancing interventions aimed at alleviating FOC.

## 2 Methods

This study was conducted in accordance with the Preferred Reporting Items for Systematic Reviews and Meta-Analyses (PRISMA) guideline (Hutton et al., 2015). The network meta-analysis was preregistered at PROSPERO (CRD42024536944).

### 2.1 Search strategy

We searched all published literature in four databases, Pubmed, Cochrane CENTRAL, and Web of Science, from the date of April 2014 to April 2024. Taking Pubmed as an example, our search strategy is “#1 Parturition [MeSH Terms] OR (Parturitions OR Birth OR Births OR Childbirth OR Childbirths OR Deliver* OR antenatal OR prenatal OR postpartum OR puerperium OR gestation OR postnatal OR Pregnan*) AND (in the last 10 years)[Filter] AND (Randomized Controlled Trial)[Filter] #2 (Fear of childbirth) OR FOC OR fear OR (fear of birth) AND (in the last 10 years)[Filter] AND (Randomized Controlled Trial)[Filter] #3 #1 AND #2.” In addition, reference lists of the included studies, and published systematic reviews and meta-analyses were hand searched to improve coverage. The specific search strategy is shown in the Supplementary material 1.

### 2.2 Inclusion criteria

We defined the target trials according to the PICOS (population, interventions, comparisons, outcomes, study design) selection criteria.

1. Population: Age > 18 years, pregnant women, not pregnancy after infertility treatment, no high-risk pregnancy conditions, no clear indication for cesarean section; normal cognitive functioning, can read and write, no other clearly diagnosed illness or mental disorder.
2. Interventions: Non-pharmacological interventions that can improve pregnant women’s fear of childbirth.
3. Comparisons: Placebo, care as usual, antenatal education (lectures, online antenatal courses, online counseling, psycho-education).
4. Outcomes: Primary outcome indicator FOC must have been assessed, otherwise studies not included. Primary outcomes include improvement of pregnant women’s FOC [measurements including W-DEQ (Wijma Delivery Experience/Expectation Questionnaire), CAQ (Childbirth Attitude Questionnaire), FOBS (Fear Of Childbirth Scale), PRAQ (Pregnancy Related Anxiety Questionnaire)]. Secondary outcomes include mode of delivery (cesarean section, natural birth), depression, anxiety, stress, childbirth self-efficacy.
5. Type of included studies: RCT (Randomized Controlled Trail).

### 2.3 Exclusion criteria

(1) Not RCT, (2) conference papers, abstracts, meta-analyses, or systematic reviews, (3) papers with incomplete data, (4) papers published not in English.

### 2.4 Data extraction

Endnote X9.3.3 was used to manage the literature. Duplicates were excluded using the duplicate literature filtering function of the software, and two authors (ZHOU & ZHU) independently screened the papers based on study titles and abstracts to obtain papers that met the criteria. We then independently extracted data from the included studies. Disagreements were resolved by discussion between the two reviewers, and a third reviewer (LI) was consulted if necessary. If a consensus could not be reached, predefined tiebreaker criteria, such as prioritizing the most recent or methodologically rigorous study, were applied to ensure an objective resolution.

During data extraction, the primary outcome measure was FOB scales and the secondary outcome measures were mode of delivery, depression, stress, anxiety and delivery self-efficacy scales. Details of the extracted data included RCT registration number, first author, year of publication, country, sample size, baseline characteristics of participants (mean age and gestational week), interventions, days of duration, outcome measure indicators. Outcome data included baseline and endpoint data on FOB, depression, stress, anxiety, and childbirth self-efficacy scales, including means and standard deviations. In addition, the number of people who chose different modes of delivery was extracted. If the outcome of a trial included several post-intervention and follow-up scores, only the score immediately after the end of the intervention phase was used.

### 2.5 Quality assessment

Two reviewers (ZHOU & ZHU) independently examined the methodological quality of individual studies by using Cochrane risk of bias tool 2.0. The assessment domains included: bias arising from the randomization process, bias due to deviations from intended interventions, bias due to missing outcome data, bias in measurement of the outcome, bias in selection of the reported result, and overall bias. The overall risk of bias judgement would be “high risk,” “low risk,” or “unclear,” which could be obtained based on signaling questions and corresponding algorithms. Any discrepancy was resolved by discussion and made a consensus.

### 2.6 Data synthesis and analysis

The network meta-analysis (NMA) was conducted using Stata (version 17) based on frequentist principles. For studies reporting outcomes as pre- and post-intervention measures, we applied methods from the Cochrane Handbook to calculate the mean change and its corresponding standard deviation (SD). Continuous outcome indicators were analyzed using the standardized mean difference (SMD) with a 95% confidence interval (95% CI) as the effect size. The incidence of cesarean section (CS) was expressed as an odds ratio (OR) with a 95% CI. Statistical significance was defined as a *p*-value of less than 0.05.

Global inconsistency tests and node-splitting tests were used to assess inconsistency. A consistency model was used only if neither of the two reported significant inconsistency (*p* > 0.05). If inconsistency was reported in any network, sensitivity analysis was performed to identify the source of inconsistency, and the study was excluded from the network. Network diagrams of the interventions were constructed to show the correlation between treatment options. Publication bias within each network was assessed using funnel plots. The relative effectiveness of interventions was ranked by the surface under the cumulative ranking curve (SUCRA). Funnel plots were used to detect potential publication bias and small-sample effects. To compare the effectiveness of non-pharmacological interventions for reducing cesarean section rates. Between-study heterogeneity was evaluated using the Cochrane *Q* test (with significance set at *p* < 0.1) and *I*^2^ statistics (with values above 50% indicating substantial heterogeneity). If heterogeneity was not significant, a fixed-effects model was used for statistical analysis; otherwise, a random-effects model was applied. To assess the effectiveness of non-pharmacological interventions in reducing fear of childbirth among primiparous women and those with severe FOC (W-DEQ-A score ≥ 85), subgroup analysis was performed.

## 3 Results

### 3.1 Literature search and screening process

**Figure 1:**
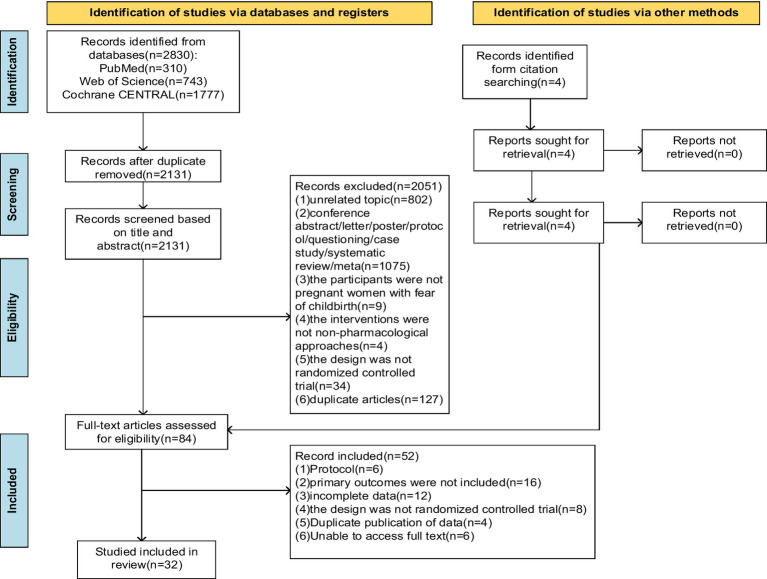
Literature screening flow chart.

A total of 2,830 articles were yielded by the search by keywords. An additional 4 articles were identified by searching the reference lists of the included studies. After eliminating 699 duplicate articles and screening the titles and abstracts, we retained 84 articles for full-text reading and review. Finally, 32 (Abdollahi et al., 2020; Alivand et al., 2023; Andaroon et al., 2017; Baas et al., 2023; Boz et al., 2021; Calpbinici and Yücel, 2022; Çankaya and Şimşek, 2021; Dai et al., 2021; Duncan et al., 2017; Emadi et al., 2023; Ghasemi et al., 2018; Gökbulut et al., 2024; Gönenç and Dikmen, 2020; Güney et al., 2022; Güven Santur and Özşahin, 2024; Kuo et al., 2022; Lee et al., 2023; Lida and Fariborz, 2017; Mortazavi and Mehrabadi, 2021; Navaee and Abedian, 2015; Ozbek and Pinar, 2022; Rondung et al., 2018; Rouhe et al., 2015; Sun et al., 2021; Thijssen et al., 2023; Toohill et al., 2017; Uludağ et al., 2022; Vakilian et al., 2023; Veringa-Skiba et al., 2022; Wang et al., 2023; Wilczyńska et al., 2023; Yesildag and Golbasi, 2024) articles were included in this meta-analysis. The study selection flow chart is shown in Figure 1.

### 3.2 Basic characteristics of included studies

The trials included a total of 3,187 pregnant women receiving 17 non-pharmacological interventions. The specific interventions are as follows: (1) motivational interview (MI) (*n* = 3), (2) high intensity interval training (*n* = 1), (3) prenatal education program (*n* = 11), (4) emotional freedom technique (EFT) (*n* = 2), (5) mindfulness-based interventions (*n* = 6), (6) deep relaxation exercise (*n* = 1), (7) acceptance and commitment therapy (*n* = 1), (8) breathing awareness training (*n* = 1), (9) haptonomy (*n* = 2), (10) CBT (*n* = 3), (11) simulation-based childbirth education (*n* = 3), (12) eye movement desensitization and reprocessing therapy (*n* = 1), (13) counseling therapy (*n* = 3), (14) dance and music therapy (*n* = 1), (15) music therapy (*n* = 1), (16) placebo (*n* = 6), (17) care as usual (*n* = 19). The table of basic characteristics of included literature can be found in Supplementary material 2.

### 3.3 Methodological quality of the studies

In general, the randomized controlled trials included in our network meta-analysis showed an acceptable and relatively low risk of biases. Almost all the included trials reported randomization but did not describe the allocation concealment in sufficient detail and were judged to have an unclear risk bias. Seven studies were judged to have a high risk of bias with regard to “blinding of participants and personnel” domain. Six studies had a high risk of bias concerned in the “blinding of outcome assessment” domain. Most studies described in detail the loss of follow-up and excluded data. Most studies had an unclear risk of selective reporting bias. Other risk of bias was not identified in the included studies. Supplementary material 3 presents the results of the methodological quality evaluation of the included studies.

### 3.4 Analyses of outcomes

Separate network meta-analyses were conducted to evaluate improvements in FOB both immediately post-intervention (during the gestational period) and after delivery. During the gestational period, FOB was measured using the W-DEQ-A, CAQ, FOBS, PRAQ-R2, and the Childbirth Self-Efficacy Questionnaire (fear subscale). In the postnatal period, the W-DEQ-B and FOBS were used as measurement instruments.

#### 3.4.1 FOB during gestational period

Overall, 32 randomized controlled trials comprising of 2,765 participants assessed the effects of 17 non-pharmacological interventions on FOB during gestational period.

**Figure 2:**
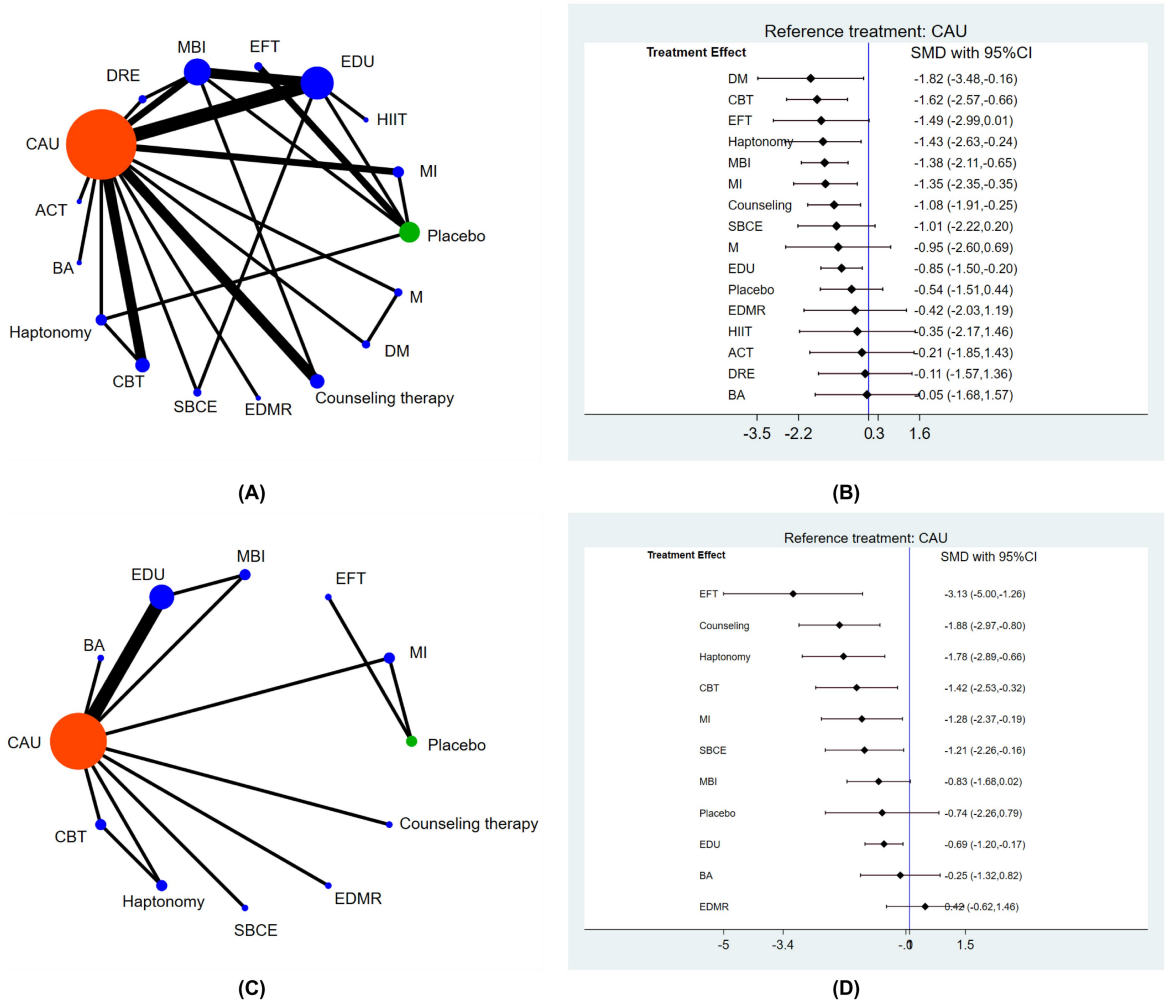
The main results of the network meta-analyses. **(A)** Network diagram of non-pharmacological interventions for fear of childbirth in the gestational period, **(B)** forest plot of pairwise comparisons between non-pharmacological interventions and usual care for reducing fear of childbirth in the gestational period, **(C)** network diagram of non-pharmacological interventions for fear of childbirth in the postnatal period, **(D)** forest plot of pairwise comparisons between non-pharmacological interventions and usual care for reducing fear of childbirth in the postnatal period. CAU = care as usual, ACT = acceptance commitment therapy, BA = breathing awareness training, CBT = cognitive behavior therapy, SBCE = simulation-based childbirth education, EDMR = eye movement desensitization and reprocessing therapy, DM = dance and music therapy, M = music therapy, MI = motivational interview, HIIT = high intensity interval training, EDU = prenatal education program, EFT = emotional freedom technique, MBI = mindfulness-based intervention, DRE = deep relaxation exercise.

The main results of the network meta-analyses are shown in Figure 2. All interventions were compared pairwise. Compared with usual care, seven non-pharmacological interventions were statistically significantly effective in reducing FOB during pregnancy. These included dance and music therapy (SMD = −1.82, 95%CI –3.48 to −0.16), CBT (SMD = −1.62, 95%CI –2.47 to −0.66), haptonomy (SMD = −1.43, 95%CI –2.63 to −0.24), mindfulness-based intervention (SMD = −1.38, 95%CI –2.11 to −0.65), MI (SMD = −1.35, 95%CI –2.35 to −0.35), counseling therapy (SMD = −1.08, 95%CI –1.91 to −0.25), antenatal education program (SMD = −0.85, 95%CI –1.50 to −0.20). No statistically significant differences were found in the pairwise comparisons of the other interventions.

A larger area in the SUCRA ranking chart for each intervention indicated a better intervention efficacy. The top three interventions were dance and music therapy (80.9), CBT (79.2) and EFT (72.6), as shown in Supplementary material 4.

#### 3.4.2 FOB during postnatal period

Overall, 14 randomized controlled trials comprising of 1,388 participants assessed the effects of 12 non-pharmacological interventions on FOB during postnatal period.

The main findings of the network meta-analyses are shown in Figure 2. All intervention measures were compared pairwise. Compared with usual care, seven non-pharmacological interventions were statistically significantly effective in reducing fear of childbirth during postnatal period. They included EFT (SMD = −3.13, 95%CI –5.00 to −1.26), counseling therapy (SMD = −1.81, 95%CI –2.97 to −0.80), haptonomy (SMD = −1.78, 95%CI –2.89 to −0.66), CBT (SMD = −1.42, 95%CI –2.53 to −0.32), MI (SMD = −1.28, 95%CI –2.37 to −0.19), stimulate-based childbirth education (SMD = −1.21, 95%CI –2.26 to −0.16), antenatal education program (SMD = −0.69, 95%CI –1.20 to –0.17). No statistically significant differences were found in the pairwise comparisons of any other interventions.

According to the SUCRA ranking chart, the top three interventions were EFT (96.6), counseling therapy (79.4) and haptonomy (77.3), as shown in Supplementary material 4.

#### 3.4.3 Subgroup analysis

Seven randomized controlled trials comprising of 316 participants assessed the effects of 9 non-pharmacological interventions on FOB for primigravida during gestational period. All intervention measures were compared pairwise. Compared with placebo, three interventions were statistically significantly effective in reducing FOB of primigravida. They included MI (SMD = −0.57, 95%CI –1.04 to −0.11), EFT (SMD = −1.13, 95%CI –1.52 to −0.73), haptonomy (SMD = −1.21, 95%CI –2.26 to −0.16). Compared with usual care, two interventions including counseling therapy (SMD = −0.89, 95%CI –1.27 to −0.52) and simulate-based childbirth education (SMD = −0.88, 95%CI –1.43 to −0.33) were statistically significantly effective. The top intervention was EFT (SUCRA value: 98.8).

Nine randomized controlled trials comprising of 1,090 participants assessed the effects of 8 non-pharmacological interventions on severe fear of childbirth during gestational period. Compared with usual care, four interventions including CBT (SMD = −1.50, 95%CI –2.78 to −0.22), haptonomy (SMD = −1.67, 95%CI –2.95 to −0.38), mindfulness-based interventions (SMD = −1.58, 95%CI –3.01 to −0.16) and antenatal education program (SMD = − 1.15, 95%CI –1.89 to −0.41) were statistically significantly effective. The top intervention was haptonomy (SUCRA value: 77.9). The main results of subgroup analyses are shown in Supplementary material 4.

#### 3.4.4 Cluster analysis

**Figure 3:**
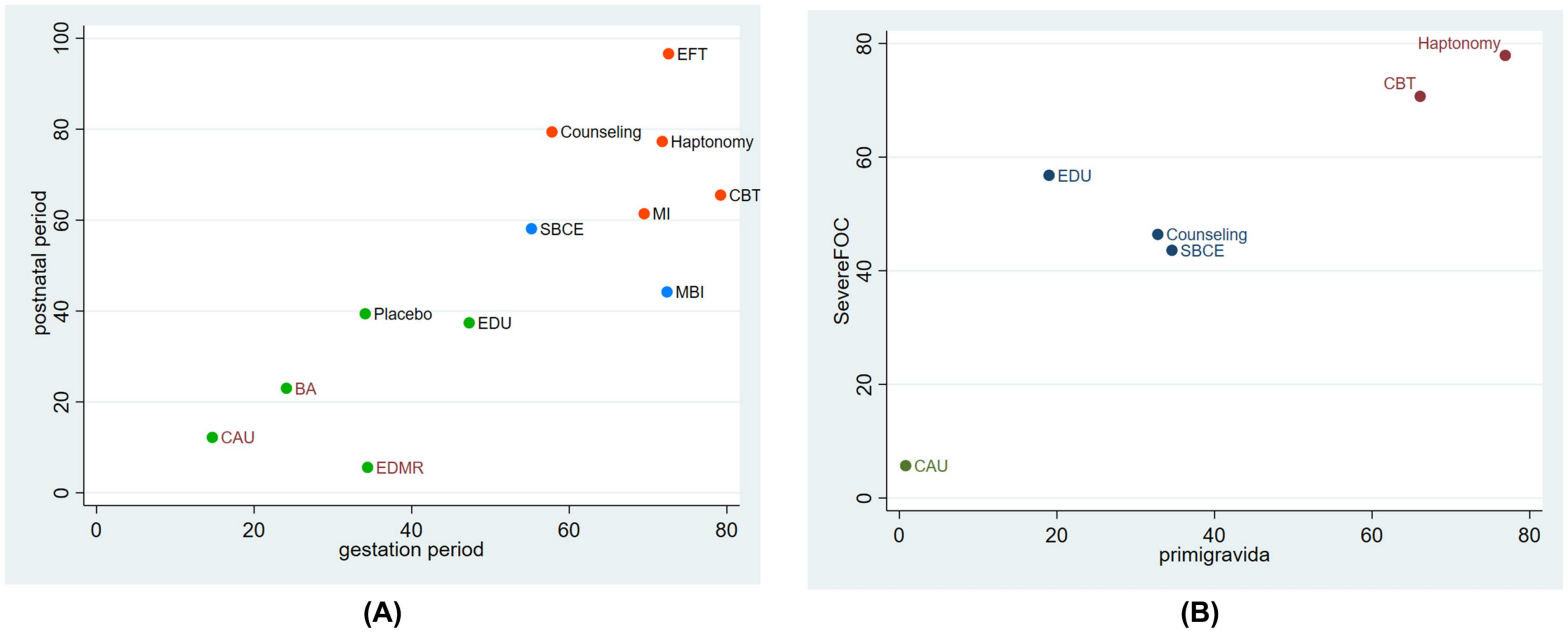
The results of Cluster analyses. **(A)** Cluster analysis plot of the effect of non-pharmacological interventions on fear of childbirth in the gestational and postnatal period, **(B)** cluster analysis of the effectiveness of non-pharmacological interventions on fear of childbirth among primiparous women and those with severe childbirth fear.

To better analyze the efficacy of different interventions in improving FOC during both gestational and postnatal periods and recommend more appropriate interventions for pregnant women, a cluster analysis was performed to combine the two outcome indicators to give a holistic evaluation. The different interventions were labeled using different colors. The more to the upper right the intervention, the better its efficacy, while the more to the lower left the intervention, the worse its efficacy. As shown in Figure 3, the five most effective interventions were EFT, counseling therapy, haptonomy, MI, and CBT.

We also conducted a cluster analysis of the effectiveness of different interventions among primigravida and pregnant women with severe FOC. The two most effective interventions were haptonomy and CBT.

#### 3.4.5 Depression, stress, anxiety and childbirth self-efficacy

After extracting the data on depression, stress, anxiety and childbirth self-efficacy from the included studies, the comparative effectiveness of interventions to improve these psychological symptoms was assessed by conducting pairwise meta-analyses using the random-effects model to directly compare the treatment effects of any two non-pharmacological interventions. Compared with usual care, counseling therapy was statistically significantly effective in reducing symptoms of depression, stress, and anxiety in pregnant women. No interventions are significantly better than other interventions at improving childbirth self-efficacy. Detailed results are provided in the Supplementary material 4.

#### 3.4.6 Mode of delivery

**Figure 4:**
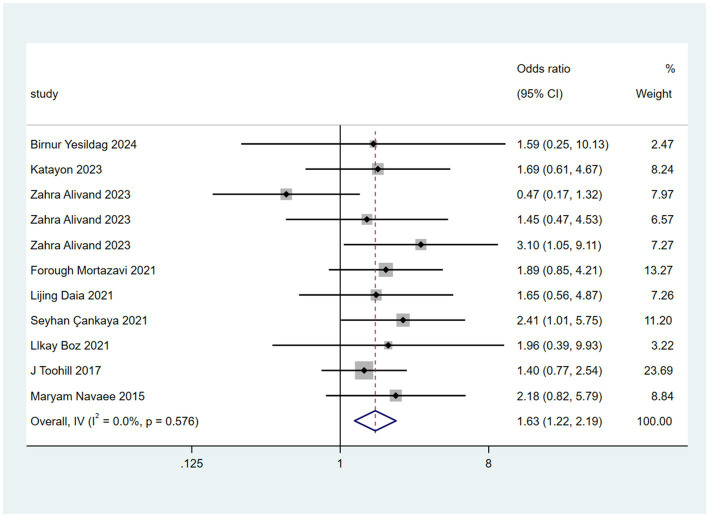
Forest plot of cesarean section rates across non-pharmacological interventions.

Nine randomized controlled trials reported on the mode of delivery. As shown in Figure 4, The *I*^2^ statistic (0.0%) and *p*-value for heterogeneity (*p* = 0.576) indicate no significant heterogeneity among studies, meaning the results are consistent across studies. Two interventions demonstrated statistically significant effectiveness in reducing the cesarean section rate: haptonomy (OR = 3.10, 95% CI 1.05–9.11) and antenatal education programs (OR = 2.41, 95% CI 1.01–5.75).

### 3.5 Heterogeneity and consistency analysis

**Figure 5:**
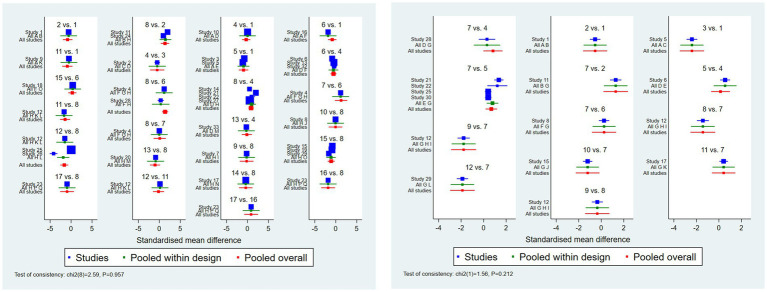
Forest plots of consistency analyses.

Inconsistency of the data was detected according to the fit of the inconsistency model and node-splitting model, with closed-loop *p* > 0.05, suggesting no significant inconsistency between the results of direct comparison and indirect comparison. The Forest plots of consistency analyses are shown in Figure 5.

### 3.6 Overall publication bias

**Figure 6:**
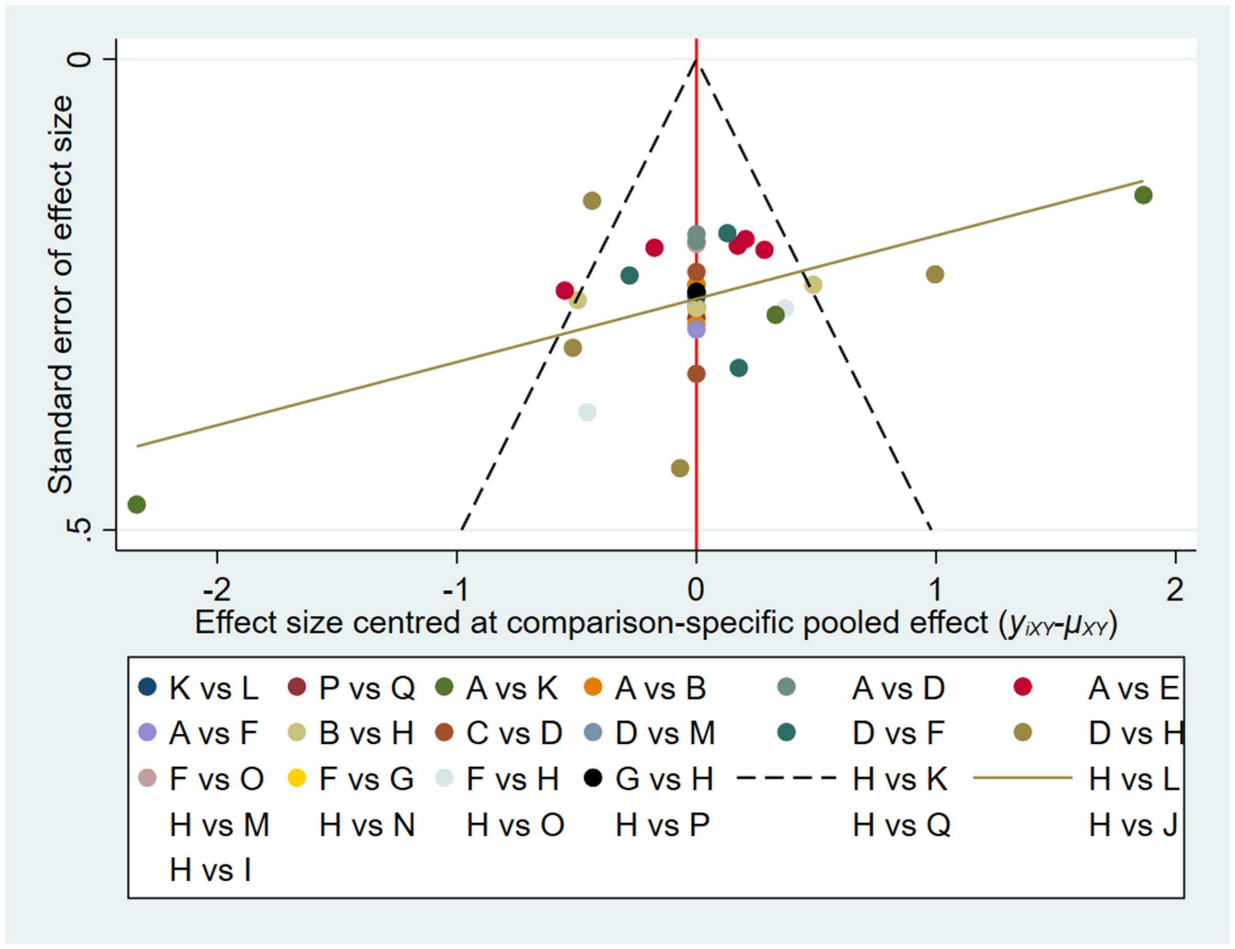
Funnel plot of overall publication bias.

As shown in Figure 6, the scatter of points on the funnel plot has shown visually symmetrical in shape around the mean estimated treatment effect, indicating low risk of publication bias.

## 4 Discussion

### 4.1 Summary

Our study included 32 randomized controlled trials, identifying 17 non-pharmacological interventions to address FOC in women through a network meta-analysis. We also extracted data on related outcomes, including depression, stress, anxiety, self-efficacy, and mode of delivery. This review demonstrated that dance and music therapy, CBT, and EFT were the top three interventions for reducing FOC. For alleviating FOC in postnatal period, EFT, counseling therapy, and haptonomy were the most effective.

Cluster analyses supported the recommendation of EFT, MI, haptonomy, counseling therapy, and CBT as effective non-pharmacological interventions for mitigating FOC. Subgroup analyses revealed that, compared with usual care, EFT was most effective for primigravida, while haptonomy had the greatest effect in women with severe FOC. Counseling therapy was found to be statistically significantly effective in reducing depression, stress, and anxiety symptoms in pregnant women compared to usual care. No single intervention showed a significant advantage over others in improving childbirth self-efficacy. However, haptonomy and antenatal education programs significantly reduced the cesarean section rate.

### 4.2 Overall completeness and applicability of evidence

The study indicates that EFT can significantly reduce FOC. EFT, which blends cognitive and exposure therapies with acupressure, uses finger tapping on acupoints and is commonly known as “tapping” (Church et al., 2022). Clinical EFT is an evidence-based treatment for PTSD, with meta-analyses supporting its efficacy for depression, anxiety, and phobias (Clond, 2016; Sebastian and Nelms, 2017; Stapleton et al., 2023). EFT is accessible, easy to train, and safe, with remote instruction proving as effective as group sessions. This scalability makes it a promising, cost-effective intervention (Church et al., 2018). However, EFT has limited research in maternal health, and further high-quality studies are warranted, particularly for women with traumatic birth histories, to confirm EFT’ s efficacy in managing childbirth-related fears.

Haptonomy is an emerging intervention that fosters a positive emotional connection between mother and fetus through touch, potentially improving maternal mental health, preventing psychiatric disorders, and promoting strong mother-infant bonding (Fernandez, 2019; Ksycinski et al., 2010). Our findings suggest that haptonomy may help alleviate severe FOC and reduce cesarean section rates. However, it remains uncertain if the reduction in cesarean rates is clinically significant, given the limited studies and the influence of factors such as medical indications, partner preferences, and socio-economic and cultural considerations on delivery choice (Sebastião et al., 2016). Additionally, current trials focused only on primiparous women, and future research should include a wider population to confirm the effectiveness of haptonomy.

MI originated in addiction treatment and fosters a partnership between clinician and patient through an encouraging and guiding conversation style. It aims to evoke motivation for change by reinforcing and exploring the patient’s reasons for change, emphasizing patient autonomy, and prioritizing their needs (Miller and Rollnick, 2015). MI has proven to be an evidence-based approach for treating substance addictions, including alcohol, marijuana, and tobacco (Bischof et al., 2021). More recently, its use has expanded to mental health areas like anxiety and eating disorders (Romano and Peters, 2015), where MI has shown greater effectiveness as an adjunct to cognitive-behavioral therapy (CBT) compared to CBT alone (Marker and Norton, 2018). In the maternal health field, MI has been less extensively applied, this study suggests that MI holds potential as a primary or adjunctive method to reduce FOC.

This study found that both CBT and counseling therapy significantly reduce FOC. These interventions are commonly used to address psychological symptoms in pregnant women. While substantial evidence supports CBT’s effectiveness in improving sleep, anxiety, and depression during pregnancy (Shortis et al., 2020; Zheng et al., 2023), its impact on FOC is less studied or yields inconclusive results (Aguilera-Martín et al., 2021). CBT includes various formats. Some studies suggest face-to-face CBT is more effective than digital CBT due to adherence challenges (Larsson et al., 2017; Rondung et al., 2018), while others find no significant differences across formats or the opposite (Yunus et al., 2022). Our study did not distinguish between CBT types, suggesting future research should explore their specific effectiveness in reducing FOC. Counseling therapy, typically led by midwives, obstetricians, or psychologists, is a traditional psychological intervention (Souto et al., 2022). Although its effectiveness in reducing FOC is well-documented, some studies report limited impacts on FOC, childbirth experiences, and cesarean rates (Larsson et al., 2015).

Each intervention has different strengths and limitations in reducing fear of childbirth. EFT significantly reduces FOC and is easy to learn, safe, and cost-effective. However, there is limited research in maternal health, particularly for women with traumatic birth histories, and more high-quality studies are needed to confirm its effectiveness. Haptonomy may improve maternal mental health and reduce CS rates. However, current studies focus only on primiparous women, and the clinical significance of reduced cesarean rates remains uncertain. MI shows potential as an effective intervention for FOC, and further research is needed to explore its role in this context. CBT and counseling therapies are commonly used to reduce FOC. However, differences between CBT formats need further exploration. Counseling therapy is also effective in reducing FOC, though its impact on CS rates and childbirth experiences may be limited, suggesting a need for more effective alternatives. Further large-scale studies are needed to compare their efficacy and guide healthcare professionals in developing more effective strategies. Future research should compare the long-term effects of these interventions in larger samples and develop clinically practical, highly sensitive FOC assessment tools for rapid screening.

### 4.3 Limitations

This study has several limitations requiring attention in future research. First, we did not conduct stratified analyses on sociodemographic factors that may influence FOC, such as age, gestational age, educational level, and income, nor did we account for variables such as treatment initiation and duration, which could introduce heterogeneity into the study. Second, some interventions, like music and dance therapy, were evaluated in only one study with small sample sizes, limiting result applicability and reliability. Third, the use of varying measurement scales with different structures, scoring criteria, and sensitivities may have introduced inconsistencies. Fourth, although all studies reported FOC outcomes, most did not assess related indicators such as depression, anxiety, stress, childbirth self-efficacy, or delivery methods. Fifth, many studies lacked adequate allocation concealment and blinding, risking bias and potential overestimation of intervention effects. Sixth, the inclusion of only English-language published literature introduces a potential risk of bias, as it may exclude relevant studies published in other languages.

Future research should compare the long-term effects of these interventions in larger samples and develop clinically practical, highly sensitive FOC assessment tools for rapid screening.

## 5 Conclusion

This study shows that EFT, haptonomy, MI, CBT, and counseling are significantly more effective than standard care in reducing prenatal and postpartum FOC. EFT was most effective for primiparous women, while haptonomy was most effective for women with severe FOC. Counseling significantly reduced depression, stress, and anxiety in pregnant women compared to usual care. No intervention showed a distinct advantage in improving childbirth self-efficacy. Based on these findings, EFT, haptonomy, MI, CBT, and counseling are recommended non-pharmacological interventions for FOC. Further research is needed to conduct high-quality investigations into the effectiveness of non-pharmacological interventions in addressing FOC.

## Data Availability

The original contributions presented in the study are included in the article/Supplementary material, further inquiries can be directed to the corresponding author.
