# Supplementary material for: Comparative efficacy of non-pharmacological interventions on fear of childbirth for pregnant women: a systematic review and network meta-analysis
Source: Front Psychol. 2025 Mar 12;16:1530311. doi: 10.3389/fpsyg.2025.1530311 (PMC11938124; doi:10.3389/fpsyg.2025.1530311)
Supplement: Supplementary file 1 [file Data_Sheet_1.PDF]

PubMed

#1 Parturition[MeSH Terms] OR (Parturitions OR Birth OR Births OR Childbirth OR Childbirths OR Deliver* OR antenatal OR prenatal OR postpartum OR puerperium OR gestation OR postnatal OR Pregnan*) AND (in the last 10 years)[Filter] AND (Randomized Controlled Trial)[Filter]

#2 (Fear of childbirth) OR FOC OR fear OR (fear of birth) AND (in the last 10 years)[Filter] AND (Randomized Controlled Trial)[Filter]

#3 #1 AND #2

Web of Science

#1 TS=(Parturition OR (Parturitions OR Birth OR Births OR Childbirth OR Childbirths OR Deliver* OR antenatal OR prenatal OR postpartum OR puerperium OR gestation OR postnatal OR Pregnan*))

#2 TS=((Fear of childbirth) OR FOC OR fear OR (fear of birth))

TI=((Randomi* Control* Trial*) OR (Control* Clinical Trial*) OR Randomi* OR Control* OR Trial*)

#3 #1 AND #2

#4 #3 AND Publication Year=(2024 OR 2023 OR 2022 OR 2021 OR 2021 OR 2020 OR 2019 OR 2018 OR 2017 OR 2016 OR 2015 OR 2014)

C0chrane Library

#1 MeSH descriptor: [Parturition] explode all trees

#2 (Parturitions OR Birth OR Births OR Childbirth OR Childbirths OR Deliver* OR antenatal OR prenatal OR postpartum OR puerperium OR gestation OR postnatal OR Pregnan*):ab (Word variations have been searched)

#3 ((Fear of childbirth) OR FOC OR fear OR (fear of birth)):ab (Word variations have been searched)

#4 #1 OR #2

#5 #3 AND #4

[Filter]Cochrane Central Register of Controlled Trials

Issue 3 of 12, March 2024
