# Supplementary material for: Comparative efficacy of non-pharmacological interventions on fear of childbirth for pregnant women: a systematic review and network meta-analysis
Source: Front Psychol. 2025 Mar 12;16:1530311. doi: 10.3389/fpsyg.2025.1530311 (PMC11938124; doi:10.3389/fpsyg.2025.1530311)
Supplement: Supplementary file 2 [file Table_1.DOCX]

**Supplemental material 2. Characteristics of included studies**

| **No.** | **Author, year** | **County(region)** | **Sample size** | | **Age(year)** | | **Gestational age(week)** | | **Interventions** | **Duration of interventions** | **Primary Outcomes** | **Secondary outcomes** | **Register information** |
| --- | --- | --- | --- | --- | --- | --- | --- | --- | --- | --- | --- | --- | --- |
|  |  |  | **E** | **C** | **E** | **C** | **E** | **C** |  |  |  |  |  |
| 1 | Birnur Yesildag^[1]^ et al., 2024 | Turkey | 37 | 37 | 18~35 | 18~35 | 28~30 | 28~30 | E:motivational interview  C:placebo | 5 weeks | W-DEQ-A, W-DEQ B | Childbirth Self-efficacy  Scale | / |
| 2 | Dominika^[2]^ et al. ,2023 | Poland | 22 | 16 | 30±4 | 30±4 | 21±4 | 23±4 | E:High Intensity Interval Training  C:prenatal education program | 8 weeks | Childbirth  Attitudes Questionnaire  (CAQ) | Beck Depression Inventory-II | ClinicalTrials.gov (NCT05009433) |
| 3 | Sinem^[3]^ et al., 2024 | Turkey | 55 | 55 | 28.40  ± 4.97 | 29.09  ±  4.78 | 6~16 | 6~16 | E:Emotional Freedom Techniques  C:placebo | 3 weeks | Pregnancy-Related Anxiety Questionnaire-R2(fear of delivery scales) | / | clinicaltrials.gov( NCT05337852) |
| 4 | Nilay^[4]^ et al., 2024 | Turkey | 32/31 | 32 | 28.5 ± 8.0 | 30.0 ± 9.0 | 24.0 ± 2.0/25.0 ± 3.0 | 24.0 ± 4.0 | E:mindfulness-based intervention/deep relaxation exercise  C:care as usual | 4 weeks | Pregnancy-Related Anxiety Questionnaire-R2(fear of delivery scales) | / | Trial registration: NCT05447000 |
| 5 | Seyedeh^[5]^ et al., 2023 | Iran | 58 | 58 | 22.14 ± 3.70 | 22.06 ± 3.78 | 29.13 ± 0.76 | 29.16 ± 0.77 | E:Emotional Freedom Techniques  C:placebo | 12 weeks | W-DEQ A, W-DEQ B | / | Iranian Registry of Clinical Trials (Ref. ID: IRCT20210622051671N1) |
| 6 | WANG^[6]^ et al., 2023 | China | 40 | 43 | 31.83 ± 3.50 | 31.51 ± 3.00 | 26.23 ± 3.80 | 27.00 ± 4.10 | E:mindfulness-based interventions  C:prenatal education program | 3 weeks | W-DEQ A, W-DEQ B | Edinburgh Postnatal  Depression Scale (EPDS), Self-Rating Anxiety Scale  (SAS), | Chinese Clinical Trial Registry(ChiCTR): ChiCTR2000033149 |
| 7 | Katayon^[7]^ et al., 2023 | Iran | 33 | 33 | 26.06 ± 4.79 | 24.55 ± 5.35 | 20~25 | 20~25 | E:f acceptance and commitment therapy  C:care as usual | 8 weeks | Childbirth  Attitudes Questionnaire (CAQ) | Mode of delivery | Clinical Trial Registration No.IRCT20190910044736N2 |
| 8 | Kirsten^[8]^ et al., 2023 | Netherlands | 43 | 36 | 32.7 ± 4.1 | 34.0 ± 4.5 | 39.6 | 39.9 | E:breathing awareness  C:care as usual | / | W-DEQ A, W-DEQ B | / | The Institutional Review Board of Máxima Medical Centre approved the protocol (NL41219.015.12) |
| 9 | Hilal^[9]^ et al., 2022 | Turkey | 36 | 36 | 27.30 ± 3.37 | 27.27 ± 2.74 | 24.27 ± 1.81 | 24.44 ± 2.26 | E:haptonomy  C:care as usual | 7 weeks | W-DEQ A | Perceived stress scale (PSS) | Clinicaltrials.gov(NCT05240092) |
| 10 | Yaelim^[10]^ et al., 2023 | Korea | 60 | 56 | 32.48  ±  3.38 | 31.07  ±  2.37 | 20.08  ±  8.44 | 20.54  ±  7.31 | E:prenatal education program  C:placebo | 4 weeks | W-DEQ A | Prenatal  Distress Questionnaire (PDQ) | Trial protocol (KCT0007805) |
| 11 | Pelin Calpbinici^[11]^ et al., 2022 | Turkey | 37 | 36 | 18~35 | 18~35 | 26.32 ± 1.65 | 26.61 ± 1.32 | E：motivational interview C:care as usual | 4 weeks | W-DEQ A, W-DEQ B | The Childbirth Self‑Efcacy Scale‑Short Form (CBSEI‑SF) | ClinicalTrials.gov (Ref. No:NCT04660981) |
| 12 | Alivand^[12]^ et al., 202^[23]^3 | Iran | 32/33 | 33 | 25.82 ± 6.12/ 25.15 ± 5.19 | 23.27 ± 5.19 | 24~28 | 24~28 | E:cognitive-behavioral therapy/haptonomy  C:care as usual | 8 weeks | W-DEQ A, W-DEQ B | Mode of delivery | Iranian Registry(IRCT20170506033834N9) |
| 13 | Irena^[13]^ K et al., 2022 | Netherlands | 57 | 56 | 33.11 ± 3.92 | 32.72 ± 3.86 | 16~26 | 16~26 | E:mindfulness-based interventions  C:prenatal education program | 10 weeks | W-DEQ A | DSM-5 Perinatal  Anxiety Disorder-Labor (DSM-5 PAD-L) | Netherlands Trial  Register(2013-CDE-3064) |
| 14 | Elif Uluda^[14]^ et al., 2022 | Turkey | 23 | 21 | 26.69 ± 4.93 | 25.66 ± 4.58 | 29.13 ± 4.90 | 27.38 ± 3.16 | E:prenatal education program  C:care as usual | 2 weeks | The fear of birth scare(FOBS) | / | ClinicalTrials.gov (NCT05115071) |
| 15 | Tzu-Chi Kuo^[15]^ et al., 2022 | Taiwan | 53 | 53 | 34 ± 4 | 33.7 ± 4.8 | 12~24 | 12~24 | E:simulation-based childbirth education C:care as usual | 8 weeks | W-DEQ A, W-DEQ B | Edinburgh Postnatal Depression Scale(EPDS), State-Trait Anxiety Inventory(STAI) | ClinicalTrials.gov registry(NCT04214431 |
| 16 | Esra Güney^[16]^ et al., 2022 | Turkey | 42 | 42 | 29.36 ± 5.72 | 30.74 ± 4.73 | 23.40 ± 6.87 | 22.33 ± 4.94 | E:mindfulness-based interventions  C:placebo | 4 weeks | Childbirth  Attitudes Questionnaire(CAQ) | Beck Anxiety Inventory(BAI), Prenatal Distress Questionnaire(PDQ) | / |
| 17 | Melanie^[17]^ et al., 2023 | Netherlands | 70 | 71 | 33.5 ± 5.0 | 34.6 ± 4.6 | 115.6 ± 23.29 days | 121.0 ± 23.1 days | E:eye movement desensitization and reprocessing therapy  C:care as usual | / | W-DEQ A | / | (www.trialregister.nl, NTR5122 |
| 18 | Yaoyao Sun^[18]^ et al., 2021 | China | 70 | 68 | 30.27 ± 3.80 | 29.55 ± 4.21 | 96.77 ± 14.054 days | 100.85 ± 15.180 days | E:mindfulness-based interventions  C:counseling therapy | 8 weeks | W-DEQ A | Edinburgh Postnatal  Depression Scale (EPDS), Patient Health Generalized Anxiety Disorder-7(GAD-7) | Chinese Clinical Trial Registry (ChiCTR1900028521) |
| 19 | Forough^[19]^ et al., 2021 | Iran | 62 | 59 | 26.24 ± 5.26 | 26.10 ± 4.88 | 27.89 ± 1.58 | 28.00 ± 0.98 | E:counseling therapy C:care as usual | 5 weeks | W-DEQ A | Anxiety Scale for Pregnancy(ASP), mode of delivery | / |
| 20 | Lijing^[20]^ et al., 2021 | China | 26 | 30 | 28.42 ± 2.53 | 28.20 ± 2.19 | 28.92 ± 1.98 | 28.57 ± 2.03 | E:simulation-based childbirth education C:care as usual | 2 weeks | W-DEQ A | Childbirth Self-Efficacy Inventory, mode of delivery | Chinese clinical trials registry(NO: ChiCTR1900025309) |
| 21 | Seyhan^[21]^ et al., 2021 | Turkey | 57 | 59 | 26.4 ± 3.1 | 25.3 ± 3.7 | 30.7 ± 5.6 | 31.7 ± 4.8 | E:prenatal education program  C:care as usual | 4 weeks | W-DEQ A, W-DEQ B | The Depression Anxiety and Stress Scale (DASS-21), mode of delivery, Childbirth Self- Efficacy Inventory (CBSEI) | ClinicalTrials.gov (ref. no: removed for blind review) |
| 22 | Llkay Boz^[22]^ et al., 2021 | Turkey | 12 | 12 | 27.67 ± 4.14 | 28.75 ± 4.71 | 22.67 ± 0.78 | 23.22 ± 0.78 | E:prenatal education program  C:care as usual | 4 weeks | W-DEQ A, W-DEQ B | Mode of delivery | / |
| 23 | Ilknur^[23]^ et al., 2020 | Turkey | 31/32 | 32 | 18~35 | 18~35 | 38~42 | 38~42 | E:dance and music therapy/only music therapy  C:care as usual | 30 mins | W-DEQ A | / | / |
| 24 | Somayeh^[24]^ et al., 2020 | Iran | 35 | 34 | 25.4 ± 5.26 | 24.47 ± 4.27 | 27.6 ± 1.78 | 28.11 ± 2.02 | E:motivational interview  C:care as usual | 5 weeks | W-DEQ A | Spielberger State-Trait Anxiety  Questionnaire, Prenatal Distress  Questionnaire(PDQ), Childbirth self-efficacy inventory(CSEI) | Iranian Registry of Clinical Trials( IRCT 20110228005931N5) |
| 25 | Elisabet^[25]^ et al., 2018 | Sweden | 127 | 131 | 29.6 ± 4.88 | 29.6 ± 4.88 | 17~20 | 17~20 | E:cognitive-behavioral therapy C:care as usual | 8 weeks | The fear of birth scare(FOBS) | / | ClinicalTrials.gov (NCT02306434) |
| 26 | Farideh Ghasemi^[26]^ et al., 2018 | Iran | 30 | 30 | 25.8 ± 4.8 | 26.8 ± 3.4 | 26.7 ± 1.0 | 26.4 ± 1.1 | E:cognitive-behavioral therapy C:care as usual | 8 weeks | Self-Efficacy Questionnaire(fear dimension) | Self-Efficacy Questionnaire(anxiety dimension) | Iranian Clinical Trial Registry(IRCT2015063022983N1) |
| 27 | J Toohill^[27]^ et al., 2017 | Australia | 91 | 93 | 28.5 | 28.7 | 24~34 | 24~34 | E:prenatal education program  C:care as usual | 10 weeks | W-DEQ A | Mode of delivery | Australian New Zealand Controlled Trials Registry ACTRN12612000526875 |
| 28 | Duncan^[28]^ et al., 2017 | USA | 15 | 14 | / | / | 31 | 31 | E:mindfulness-based interventions  C:care as usual; | 2.5 days | W-DEQ A, W-DEQ B | Childbirth  Self-Efficacy Inventory (CSEI), Epidemiologic Studies Depression  Scale(CES-D) | ClinicalTrials.gov(: NCT02327559) |
| 29 | Nafise Andaroon^[29]^ et al., 2017 | Iran | 45 | 45 | 18~35 | 18~35 | 28~30 | 28~30 | E:counseling therapy  C:care as usual | / | W-DEQ A, W-DEQ B | Edinburgh Postnatal  Depression Scale (EPDS) | IRCT2016042827653N1 |
| 30 | Rouhe^[30]^ et al., 2015 | Sweden | 107 | 177 | / | / | 21.5 ± 3.7 | 21.0 ± 3.7 | E:prenatal education program  C:care as usual | 6 weeks | W-DEQ B | / | / |
| 31 | Lida Ahmadi^[31]^ et al., 2017 | Iran | 19 | 19 | 28.27 ± 4.78 | 28.21 ± 4.57 | 27 ± 2.7 | 27 ± 2.5 | E:mindfulness-based interventions  C:prenatal education program | 8 weeks | Childbirth  Attitudes Questionnaire  (CAQ) | Spielberger State-Trait Anxiety  Questionnaire | Registry of Clinical Trials by No.IRCT2015111625066N1 |
| 32 | Maryam^[32]^ et al., 2015 | Iran | 35 | 32 | 24±4 | 24±4 | 34~36 | 34~36 | E:simulation-based childbirth education C:prenatal education program | 3 weeks | Childbirth  Attitudes Questionnaire(CAQ) | / | / |

^*^E:experimental group

C:control group

1. DEQ-A:Wijma Delivery Experience/Expectation Questionnaire (Version A)

W-DEQ-B:Wijma Delivery Experience/Expectation Questionnaire (Version B)

**Reference**

[1] YESILDAG B, GOLBASI Z. Effects of the web-based birth preparation programme and motivational interviews on primigravida women: Randomized-controlled study.[J]. Int J Nurs Pract, 2024,30(1):e13207.

[2] WILCZYŃSKA D, WALCZAK-KOZŁOWSKA T, SANTOS-ROCHA R, et al. Stress is not so bad-cortisol level and psychological functioning after 8-week HIIT program during pregnancy: a randomized controlled trial.[J]. Front Public Health, 2023,11:1307998.

[3] GÜVEN SANTUR S, ÖZŞAHIN Z. The Effects of Emotional Freedom Techniques Implemented During Early Pregnancy on Nausea-Vomiting Severity and Anxiety: A Randomized Controlled Trial.[J]. Journal of integrative and complementary medicine, 2024,30(9):858-868.

[4] GÖKBULUT N, CENGIZHAN S Ö, AKÇA E I, et al. The effects of a mindfulness-based stress reduction program and deep relaxation exercises on pregnancy-related anxiety levels: A randomized controlled trial.[J]. Int J Nurs Pract, 2024,30(5):e13238.

[5] EMADI S F, HEKMAT K, ABEDI P, et al. Effect of emotional freedom technique on the fear of childbirth in Iranian primiparous women: a randomized controlled trial.[J]. Front Psychol, 2023,14:1145229.

[6] WANG S L, SUN M Y, HUANG X, et al. Benefits of Mindfulness Training on the Mental Health of Women During Pregnancy and Early Motherhood: A Randomized Controlled Trial.[J]. Biomedical and environmental sciences : BES, 2023,36(4):353-366.

[7] VAKILIAN K, RAHMATI F, KHORSANDI M, et al. Counseling by Acceptance and Commitment Therapy Approach on Fear of Childbirth: A Randomized Clinical Trial, 2023[C].

[8] THIJSSEN K M J, KIERKELS J J M, van MEURS A, et al. Visualization of contractions: Evaluation of a new experience design concept to enhance the childbirth experience.[J]. Birth (Berkeley, Calif.), 2023,50(4):1025-1033.

[9] OZBEK H, PINAR S E. The effect of haptonomy applied to pregnant women on perceived stress, fear of childbirth, and prenatal attachment: randomized controlled experimental study.[J]. Current psychology (New Brunswick, N.J.), 2022:1-10.

[10] LEE Y, KIM S, CHOI S. Effectiveness of mobile-based intervention Self-care for Pregnant Women at Work: A randomized controlled trial.[J]. J Occup Health, 2023,65(1):e12402.

[11] CALPBINICI P, YÜCEL C. The effect of the training program provided to primiparous pregnant women through the motivational interview method on their fear of childbirth, childbirth self-efficacy, and delivery mode: a randomized controlled trial[J]. Curr Psychol, 2022,42:1-15.

[12] ALIVAND Z, NOURIZADEH R, HAKIMI S, et al. The effect of cognitive-behavioral therapy and haptonomy on fear of childbirth in primigravida women: a randomized clinical trial.[J]. Bmc Psychiatry, 2023,23(1):929.

[13] VERINGA-SKIBA I K, de BRUIN E I, van STEENSEL F J A, et al. Fear of childbirth, nonurgent obstetric interventions, and newborn outcomes: A randomized controlled trial comparing mindfulness-based childbirth and parenting with enhanced care as usual.[J]. Birth (Berkeley, Calif.), 2022,49(1):40-51.

[14] ULUDAĞ E, SERÇEKUŞ P, VARDAR O, et al. Effects of online antenatal education on worries about labour, fear of childbirth, preparedness for labour and fear of covid-19 during the covid-19 pandemic: A single-blind randomised controlled study.[J]. Midwifery, 2022,115:103484.

[15] KUO T, AU H, CHEN S, et al. Effects of an integrated childbirth education program to reduce fear of childbirth, anxiety, and depression, and improve dispositional mindfulness: A single-blind randomised controlled trial.[J]. Midwifery, 2022,113:103438.

[16] GÜNEY E, CENGIZHAN S Ö, KARATAŞ OKYAY E, et al. Effect of the Mindfulness-Based Stress Reduction program on stress, anxiety, and childbirth fear in pregnant women diagnosed with COVID-19.[J]. Complement Ther Clin, 2022,47:101566.

[17] BAAS M A M, STRAMROOD C A I, DIJKSMAN L M, et al. How safe is the treatment of pregnant women with fear of childbirth using eye movement desensitization and reprocessing therapy? Obstetric outcomes of a multi-center randomized controlled trial.[J]. Acta Obstet Gyn Scan, 2023,102(11):1575-1585.

[18] SUN Y, LI Y, WANG J, et al. Effectiveness of Smartphone-Based Mindfulness Training on Maternal Perinatal Depression: Randomized Controlled Trial.[J]. J Med Internet Res, 2021,23(1):e23410.

[19] MORTAZAVI F, MEHRABADI M. Effectiveness of Solution-Focused Counseling Therapy on Pregnancy Anxiety and Fear of Childbirth: A Randomized Clinical Trial[M]. 2021.

[20] DAI L, SHEN Q, REDDING S R, et al. Simulation-based childbirth education for Chinese primiparas: A pilot randomized controlled trial.[J]. Patient Educ Couns, 2021,104(9):2266-2274.

[21] ÇANKAYA S, ŞIMŞEK B. Effects of Antenatal Education on Fear of Birth, Depression, Anxiety, Childbirth Self-Efficacy, and Mode of Delivery in Primiparous Pregnant Women: A Prospective Randomized Controlled Study.[J]. Clin Nurs Res, 2021,30(6):818-829.

[22] BOZ İ, AKGÜN M, DUMAN F. A feasibility study of a psychoeducation intervention based on Human Caring Theory in nulliparous women with fear of childbirth.[J]. Journal of psychosomatic obstetrics and gynaecology, 2021,42(4):300-312.

[23] GÖNENÇ İ M, DIKMEN H A. Effects of Dance and Music on Pain and Fear During Childbirth.[J]. Journal of obstetric, gynecologic, and neonatal nursing : JOGNN, 2020,49(2):144-153.

[24] ABDOLLAHI S, FARAMARZI M, DELAVAR M A, et al. Effect of Psychotherapy on Reduction of Fear of Childbirth and Pregnancy Stress: A Randomized Controlled Trial.[J]. Front Psychol, 2020,11:787.

[25] RONDUNG E, TERNSTRÖM E, HILDINGSSON I, et al. Comparing Internet-Based Cognitive Behavioral Therapy With Standard Care for Women With Fear of Birth: Randomized Controlled Trial.[J]. Jmir Ment Health, 2018,5(3):e10420.

[26] GHASEMI F, BOLBOL HAGHIGHI N, MOTTAGHI Z, et al. The Effect of Group Counseling with Cognitive-Behavioral Approach on Self-Efficacy of Pregnant Women’s Choice of Vaginal Delivery[J]. Iran J Psychiatry be, 2018,In Press.

[27] TOOHILL J, CALLANDER E, GAMBLE J, et al. A cost effectiveness analysis of midwife psycho-education for fearful pregnant women - a health system perspective for the antenatal period.[J]. Bmc Pregnancy Childb, 2017,17(1):217.

[28] DUNCAN L G, COHN M A, CHAO M T, et al. Benefits of preparing for childbirth with mindfulness training: a randomized controlled trial with active comparison.[J]. Bmc Pregnancy Childb, 2017,17(1):140.

[29] ANDAROON N, KORDI M, KIMIAEI S A, et al. The effect of individual counseling program by a midwife on fear of childbirth in primiparous women.[J]. J Educ Health Promot, 2017,6:97.

[30] ROUHE H, SALMELA-ARO K, TOIVANEN R, et al. Group psychoeducation with relaxation for severe fear of childbirth improves maternal adjustment and childbirth experience--a randomised controlled trial.[J]. Journal of psychosomatic obstetrics and gynaecology, 2015,36(1):1-9.

[31] LIDA A, FARIBORZ B. The effectiveness of educating mindfulness on anxiety, fear of delivery, pain catastrophizing and selecting caesarian section as the delivery method among nulliparous pregnant women[J]. Nursing Practice Today, 2017,4(1).

[32] NAVAEE M, ABEDIAN Z. Effect of role play education on primiparous women's fear of natural delivery and their decision on the mode of delivery.[J]. Iran J Nurs Midwife, 2015,20(1):40-46.
