# Supplementary material for: Comparative efficacy of non-pharmacological interventions on fear of childbirth for pregnant women: a systematic review and network meta-analysis
Source: Front Psychol. 2025 Mar 12;16:1530311. doi: 10.3389/fpsyg.2025.1530311 (PMC11938124; doi:10.3389/fpsyg.2025.1530311)
Supplement: Supplementary file 3 [file Table_2.DOCX]

**Supplemental material 3. Risk of bias for included studies**

| **Author, year** | **Random Sequence Generation (selective bias)** | **Allocation concealment (selective bias)** | **Blinding of participants and personnel(performance bias)** | **Blinding of outcome assessment(detection bias)** | **Incomplete outcome data addressed(attrition bias)** | **Selective reporting(reporting bias)** | **other bias** |
| --- | --- | --- | --- | --- | --- | --- | --- |
| Birnur Yesildag et al., 2024 | low | unclear | low | high | low | unclear | unclear |
| Dominika et al. ,2024 | low | unclear | unclear | unclear | low | unclear | unclear |
| Sinem et al., 2024 | low | low | high | low | low | unclear | unclear |
| Nilay et al., 2024 | low | low | unclear | unclear | low | unclear | unclear |
| Seyedeh et al., 2024 | low | low | low | high | low | unclear | unclear |
| WANG et al., 2023 | low | unclear | unclear | unclear | low | unclear | unclear |
| Katayon et al., 2023 | low | low | unclear | unclear | low | unclear | unclear |
| Kirsten et al., 2023 | low | unclear | unclear | unclear | low | unclear | unclear |
| Hilal et al., 2023 | low | unclear | unclear | unclear | low | unclear | unclear |
| Yaelim et al., 2023 | low | unclear | low | unclear | low | unclear | unclear |
| Pelin Calpbinici et al., 2023 | low | unclear | unclear | unclear | low | unclear | unclear |
| Alivand et al., 2023 | low | unclear | unclear | low | low | unclear | unclear |
| Irena K et al., 2022 | low | low | high | low | low | unclear | unclear |
| Elif Uluda et al., 2022 | low | unclear | high | unclear | low | unclear | unclear |
| Tzu-Chi Kuo et al., 2022 | low | low | high | low | low | unclear | unclear |
| Esra Güney et al., 2022 | low | unclear | unclear | unclear | low | unclear | unclear |
| Melanie et al., 2023 | low | unclear | high | low | low | unclear | unclear |
| Yaoyao Sun et al., 2021 | low | low | high | low | low | unclear | unclear |
| Forough et al., 2021 | low | low | high | low | low | unclear | unclear |
| Lijing et al., 2021 | low | unclear | unclear | unclear | low | unclear | unclear |
| Seyhan et al., 2021 | low | low | low | high | low | unclear | unclear |
| Llkay Boz et al., 2021 | low | low | low | high | low | unclear | unclear |
| Ilknur et al., 2020 | low | low | low | high | low | unclear | unclear |
| Somayeh et al., 2020 | low | low | low | low | low | unclear | unclear |
| Elisabet et al., 2018 | low | unclear | unclear | unclear | low | unclear | unclear |
| Farideh Ghasemi et al., 2018 | low | unclear | unclear | unclear | low | unclear | unclear |
| J Toohill et al., 2017 | low | unclear | unclear | unclear | low | unclear | unclear |
| Duncan et al., 2017 | low | unclear | unclear | unclear | low | unclear | unclear |
| Nafise Andaroon et al., 2017 | low | unclear | unclear | unclear | low | unclear | unclear |
| Rouhe et al., 2015 | low | low | unclear | unclear | low | unclear | unclear |
| Lida Ahmadi et al., 2017 | low | unclear | low | unclear | low | unclear | unclear |
| Maryam et al., 2017 | low | low | low | high | low | unclear | unclear |
