# Supplementary material for: Comparative efficacy of non-pharmacological interventions on fear of childbirth for pregnant women: a systematic review and network meta-analysis
Source: Front Psychol. 2025 Mar 12;16:1530311. doi: 10.3389/fpsyg.2025.1530311 (PMC11938124; doi:10.3389/fpsyg.2025.1530311)
Supplement: Supplementary file 4 [file Table_3.DOCX]

**Supplementary material 4**

**Table 1.**SUCRA value


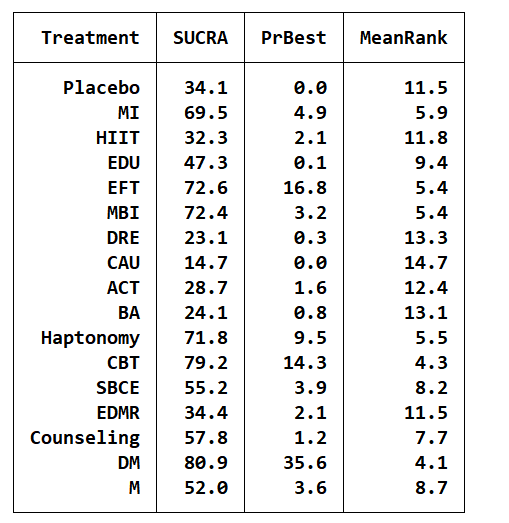

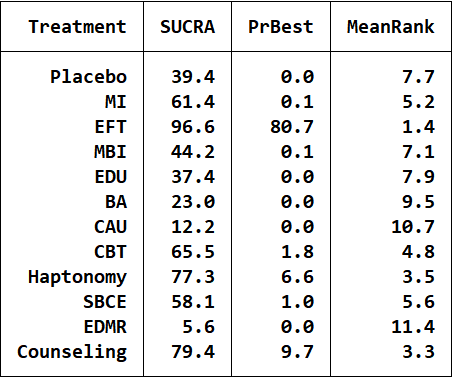


1. FOC in gestational period (b)FOC in perinatal period


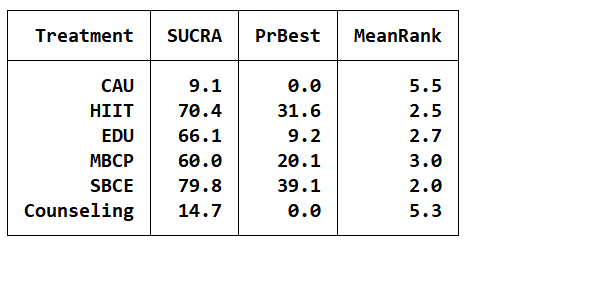

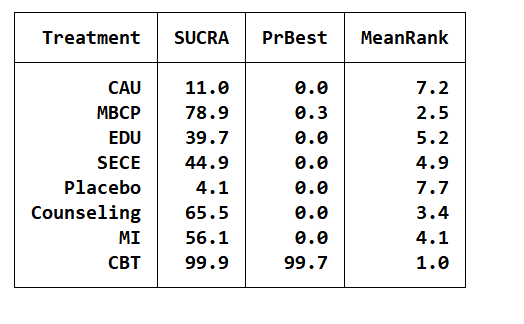


(c)depression (d)anxiety


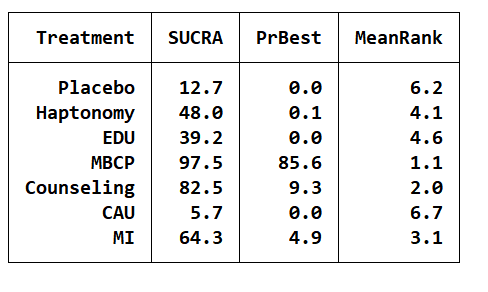

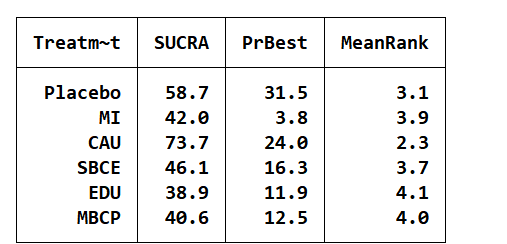


(e)stress (f)childbirth self-efficacy


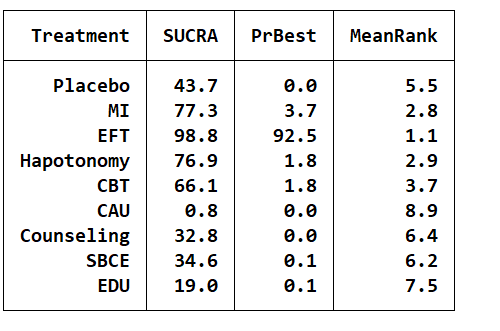

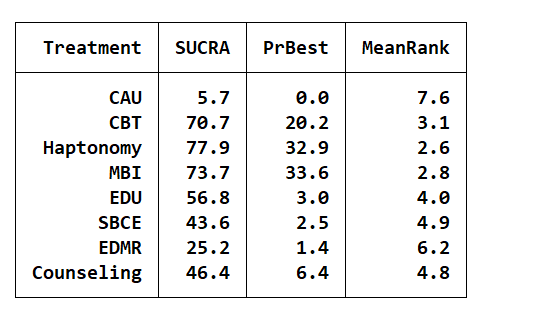


(g) FOC of primiparous (h)severe FOC

**Figure 1. SUCRA Ranking**

**
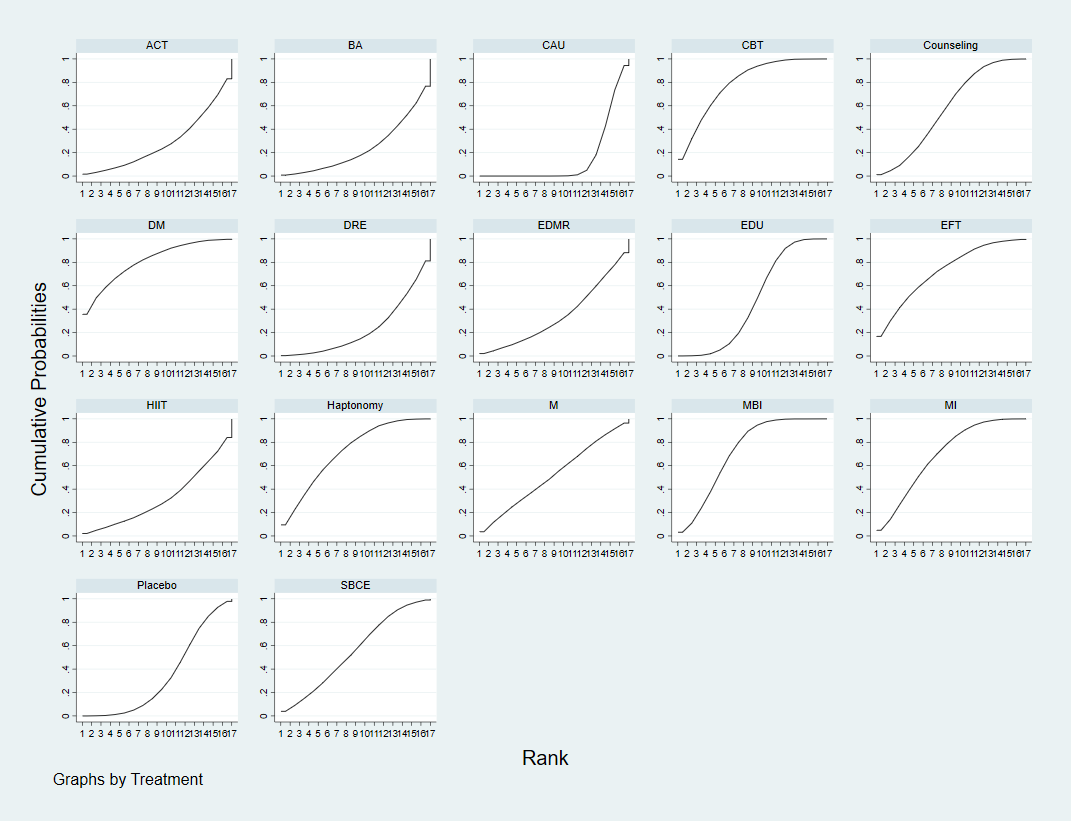
**

1. FOC in gestational period

**
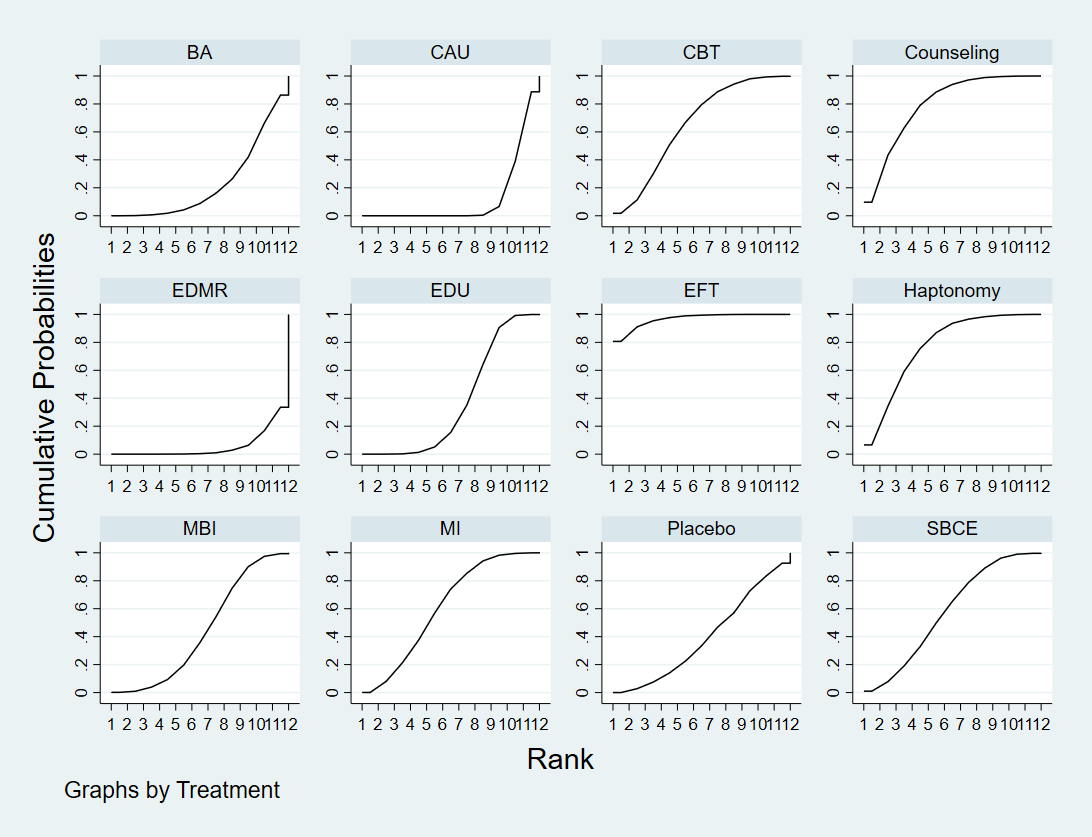
**

1. FOC in perinatal period

**Figure2. Forest plot of pairwise comparisons**

**
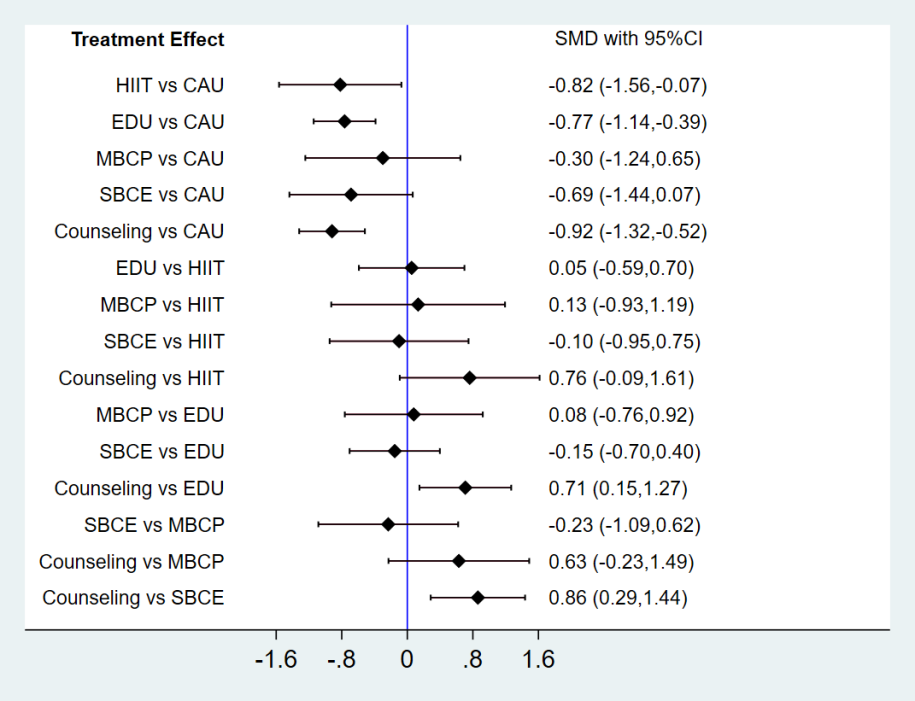
**

**(a)Depression**

**
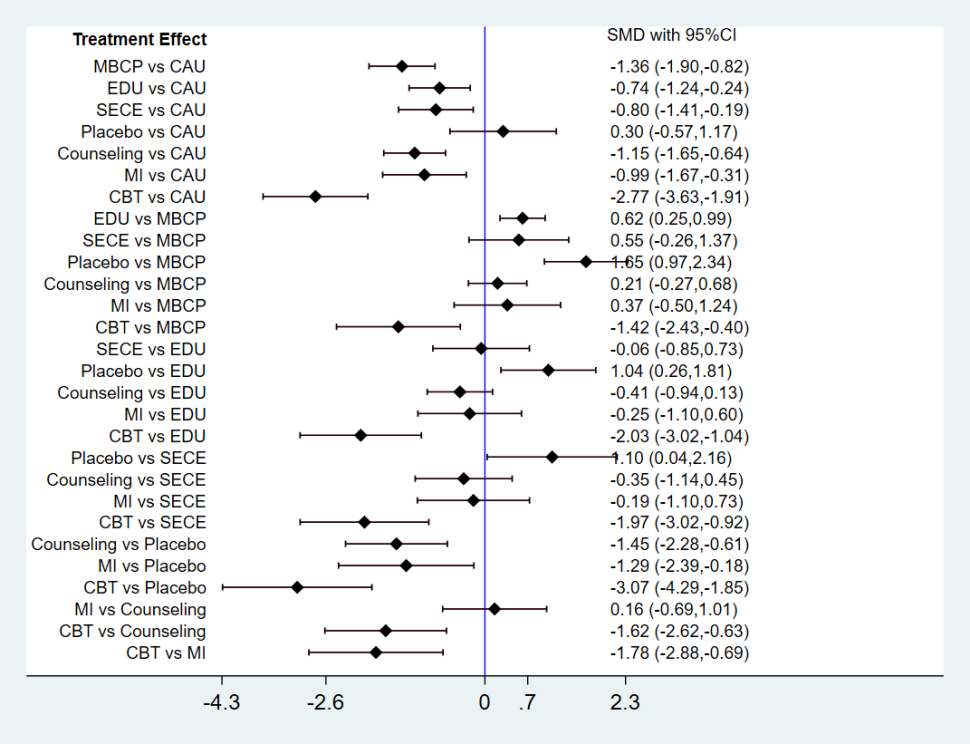
**

**(b)anxiety**

**
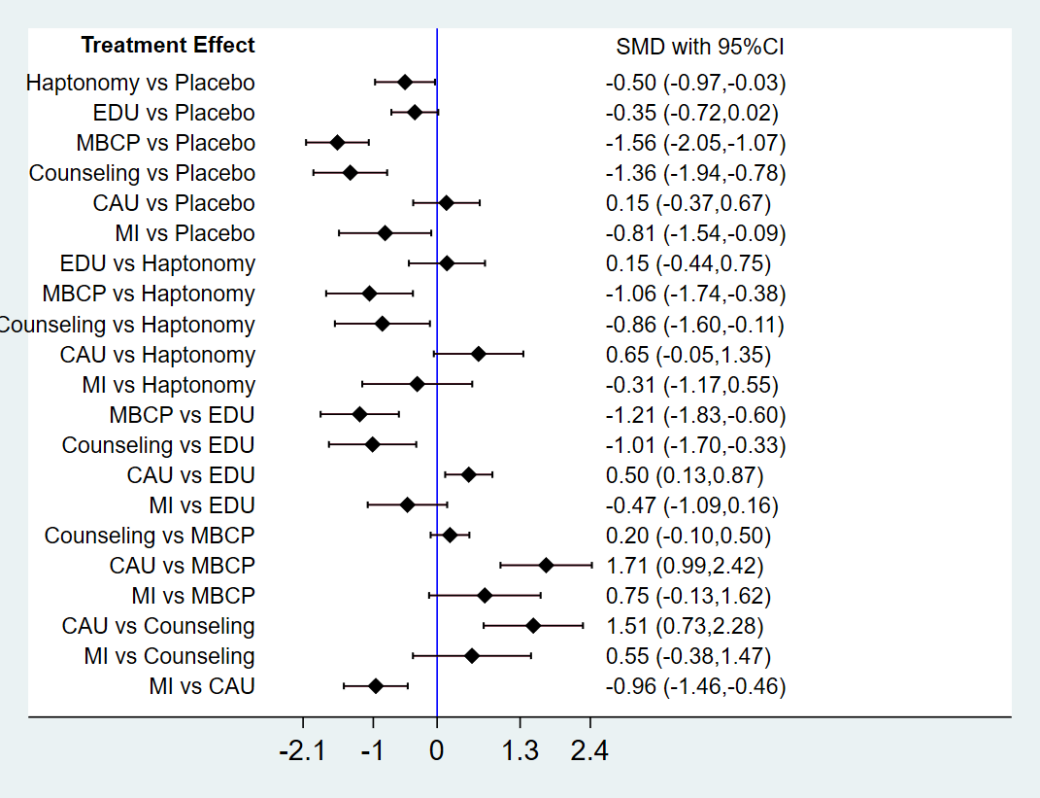
**

**(c)stress**

**
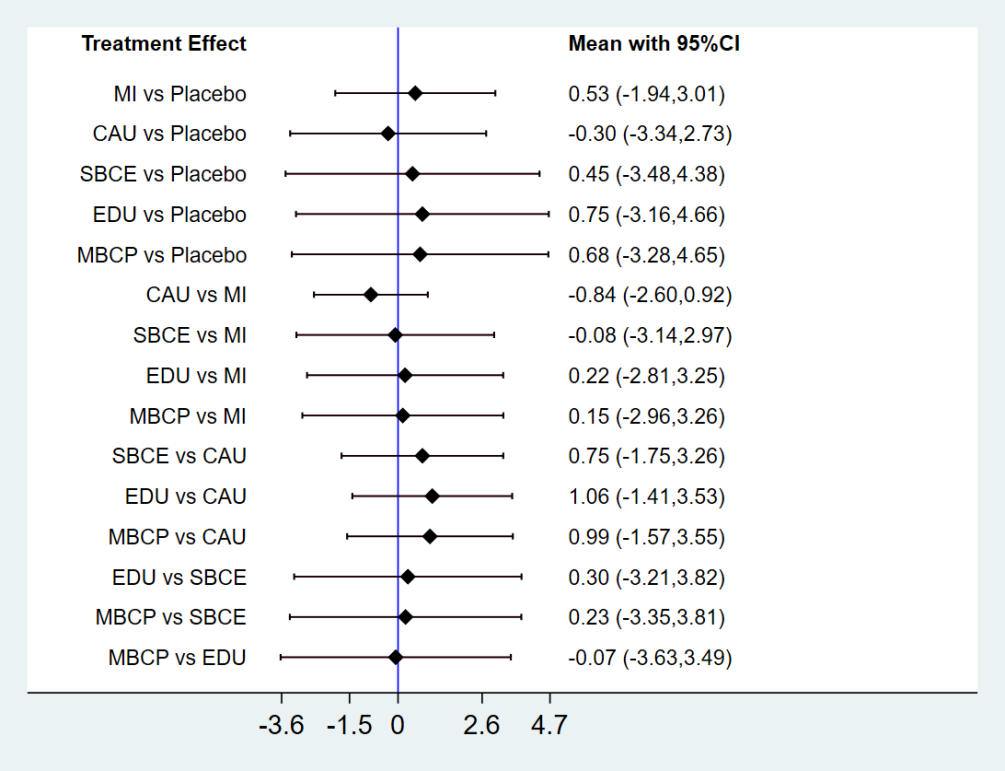
**

**(d)childbirth self-efficacy**

**
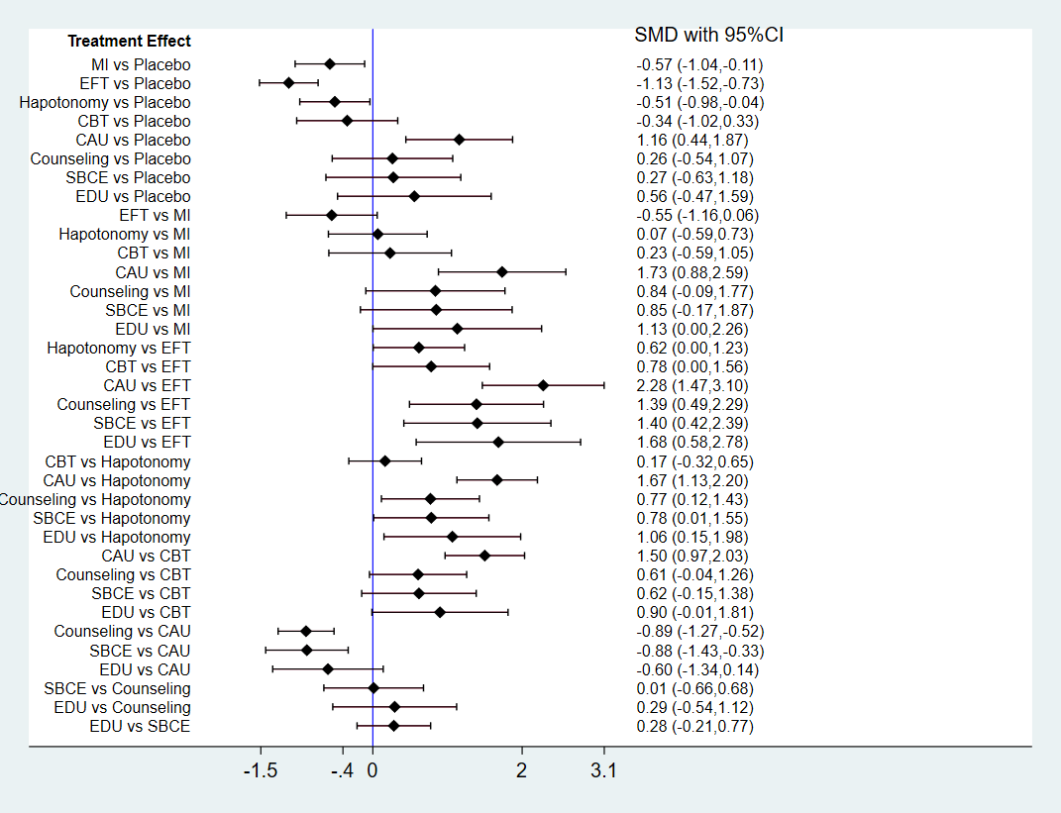
**

**(e) FOC of primiparous**

**
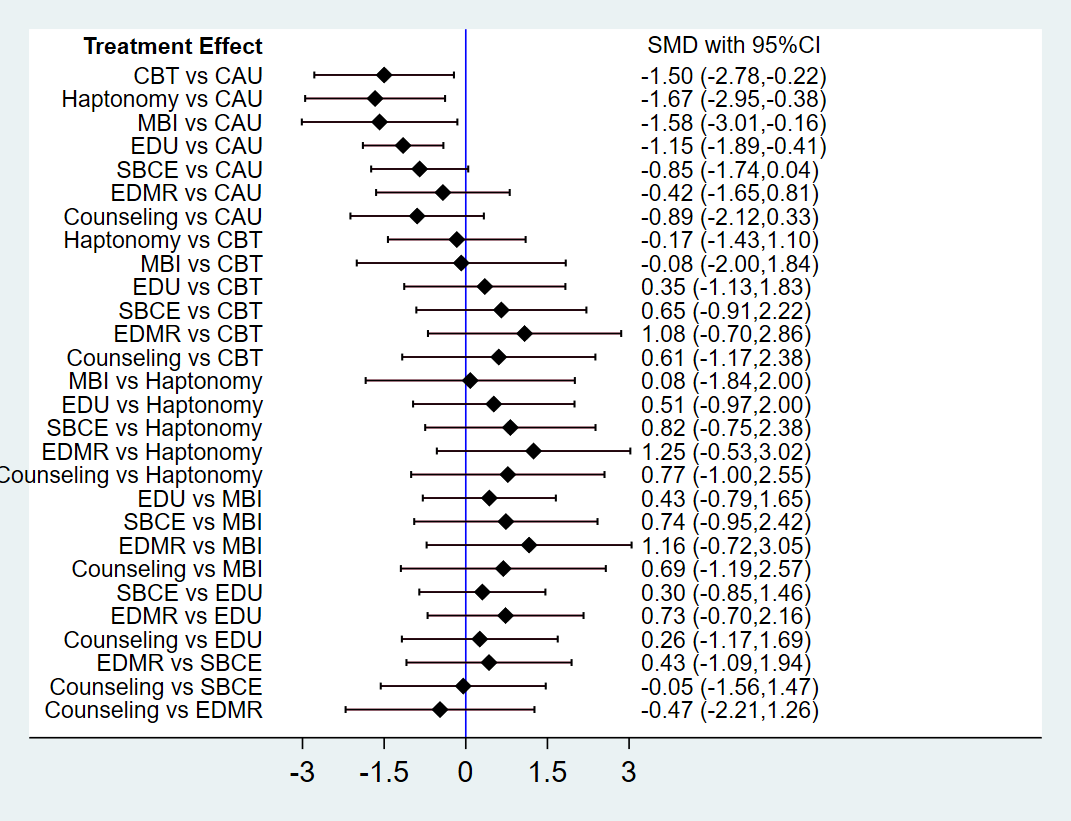
**

**(f)severe FOC**

*****CAU=care as usual, ACT=acceptance commitment therapy, BA=breathing awareness training, CBT=cognitive behavior therapy, SBCE=simulation-based childbirth education, EDMR=eye movement desensitization and reprocessing therapy, DM=dance and music therapy, M=music therapy, MI=motivational interview, HIIT=high intensity interval training, EDU=prenatal education program, EFT=emotional freedom technique, MBI=mindfulness-based intervention, DRE=deep relaxation exercise
